# COVID-19 and impairment of mental health: public health perspective

**DOI:** 10.4314/ahs.v21i4.5

**Published:** 2021-12

**Authors:** Saurabh RamBihariLal Shrivastava, Prateek Saurabh Shrivastava

**Affiliations:** 1 Medical Education Unit Coordinator and Member of the Institute Research Council, Department of Community Medicine, Shri Sathya Sai Medical College & Research Institute, Sri Balaji Vidyapeeth (SBV) - Deemed to be University, Ammapettai, Nellikuppam, Chengalpet District, Tamil Nadu - 603108; 2 Department of Community Medicine, Shri Sathya Sai Medical College & Research Institute, Sri Balaji Vidyapeeth (SBV) – Deemed to be University, Ammapettai, Nellikuppam, Chengalpet District, Tamil Nadu – 603108

**Keywords:** COVID-19 pandemic, Mental health, Depression

## Abstract

**Objectives:**

The objective of the article is to understand the impact of COVID-19 on the mental health of the members of the general population.

**Introduction:**

The coronavirus disease-2019 (COVID-19) pandemic has forced the public health authorities to implement unprecedented public health measures with an intention to control the spread of the infection.

**Methods:**

An extensive search of all materials related to the topic was carried out in the PubMed search engine and World Health Organization website and a total of 27 articles were selected based upon the suitability with the current review objectives.

**Results:**

In order to reduce the caseload and interrupt the chain of transmission of the novel viral infection, it was envisaged that people should stay indoors unless it is extremely essential. This intervention did play its part in reducing the caseload, but significantly affected the daily routine of the people, which in turn accounted for a significant impact on the mental health of the people. Considering the ongoing development and the impact of COVID-19 infection on the mental health of people, there is an immense need to implement strategies to improve the lifestyle of the general population and the health care professionals.

**Conclusion:**

To conclude, the ongoing COVID-19 pandemic has created a state of public health emergency on the global scale. The infection has impacted people from all walks of life and is also responsible for precipitating a number of psychological and mental disorders. Thus, it is the need of the hour to identify those individuals who are prone to psychological disorders, and take urgent steps to ensure the preservation and improvement of the mental health of people.

## Introduction

The coronavirus disease-2019 (COVID-19) pandemic has forced the public health authorities to implement unprecedented public health measures with an intention to control the spread of the infection.^1^ However, regardless of the multiple interventions, as on 13 July 2021, a cumulative total of 186240393 cases and 4027861 deaths, amounting to a case fatality rate of 2.16% has been reported.^2^ Even though the case fatality rate of the COVID-19 infection was lower when compared with Ebola virus disease, owing to the international spread of the infection and that too at a rapid pace, the impact of infection on mental health of the general population cannot be ignored.^3^ The purpose of the current review was to explore the impact of COVID-19 on the mental health of the general population.

## Methods

**Figure 1:**
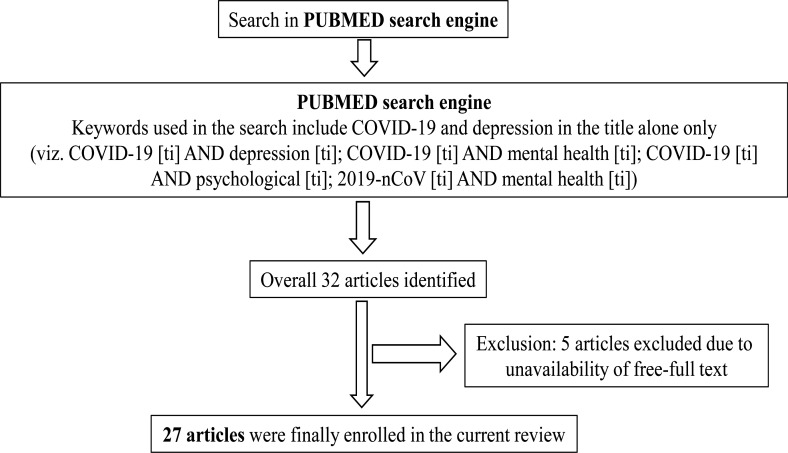
Flowchart for selection of studies

An extensive search of all materials related to the topic was carried out in the PubMed search engine and World Health Organization website. Relevant research articles focusing on COVID-19 and Obesity published in the period March 2020 to November 2020 were included in the review. A total of 32 studies similar to current study objectives was identified initially, of which, five were excluded due to the unavailability of the complete version of the articles. Overall, 27 articles were selected based upon the suitability with the current review objectives and analyzed. Keywords used in the search include COVID-19 and depression in the title alone only (viz. COVID-19 [ti] AND depression [ti]; COVID-19 [ti] AND mental health [ti]; COVID-19 [ti] AND psychological [ti]; 2019-nCoV [ti] AND mental health [ti]). The articles published in only English language were included in the current review (Figure 1). The collected information is presented under the following sub-headings, namely COVID-19 and genesis of mental health concerns, COVID-19 and impairment of mental health, Aftereffects of COVID-19 on mental health, COVID-19 and Depression, COVID-19 and depression among healthcare workers, Public health interventions, Implications for practice and Implications for research.

## COVID-19 and genesis of mental health concerns

In order to reduce the caseload and interrupt the chain of transmission of the novel viral infection, it was envisaged that people should stay indoors unless it is extremely essential.^1^,^4^ In-fact, strategies like restrictions on the mobility of people, closure of educational institutions, strategies to encourage physical distancing (viz. closure of malls, restrictions on visits to holy places, cancellation of sporting events, etc.) and declaration of health emergencies (including imposition of lockdown) in a number of the affected nations was implemented.^1^,^3^ This intervention did play its part in reducing the caseload, but significantly affected the daily routine of the people, which in turn accounted for a significant impact on the mental health of the people.^4^

## COVID-19 and impairment of mental health

The impact on mental health was due to a wide range of factors, namely the apprehension of acquisition of the infection (which has accounted for deaths of millions of people and has no cure at present), death of the loved ones, uncertainty regarding when the infection will be effectively contained, loss of jobs, or when the vaccine would be available for the use of common man.^5^–^9^ Moreover, people found the situation extremely difficult to adapt, especially considering the circumstances which were present. For instance, contacts of the COVID-19 patients or travelers were quarantined for 2 weeks where they cannot visit or meet their friends and family members and there was lots of fear and anxiety.^5^,^6^

Further, people were not aware about the psychological coping methods and they were even not having much awareness about the how and why to maintain optimal mental health.^6^ The problem was further compounded by the ongoing infodemic through different channels of social media, wherein multiple myths and misconceptions about the infection were being circulated. Whether it was multimedia or print media or social media, for months together, everything was about COVID-19 throughout the day and while being at home, many people kept watching the same and got significantly affected on the mental front.^6^,^7^ We cannot undermine the fact that there was a lack of clarity about the different aspects of the novel viral infectious disease and the ways to prevent the spread of the infection, and that further added to the anxiety & fear.^5^,^7^

## Consequences of COVID-19 on mental health

The people while being at home reported change in the sleeping pattern and found it difficult to concentrate in work while being at home.^4^,^8^ However, the people who were part of the unorganized sector, could not bear the load of lockdown and found it extremely difficult to sustain their livelihood and quality of life.^3^–^6^ The people were affected to such an extent that they committed suicide as they were unable to cope with the pressure and reality of COVID-19 pandemic.^9^,^10^ In short, the COVID-19 pandemic did not impact people on the physical health front, but had significant impact on the mental well-being.^3^–^8^ COVID-19 has been linked with a wide range of mental health problems like anxiety, depression, insomnia, somatization, social phobia, post-traumatic stress disorders, obsessive-compulsive disorders, self-harm, and suicidal ideations in different population groups like college going students or health care professionals.^11^,^12^ The findings of a systematic review reported higher rates of anxiety, depression and psychological distress among the general population affected by COVID-19 in different nations.^13^ Another systematic review and meta-analysis targeting different coronavirus revealed that 15–60% of the infected persons tend to experience serious neuropsychiatric concerns during the period of their illness, and the percentage rises even further after recovering from the infection.^14^

## COVID-19 and Depression

In general, depression is one of the commonest mental disorder accounting for disability, impairment in the quality of life and is a crucial factor towards the global burden of disease. In-fact, depression has been reported in excess of 264 million individuals distributed across the world and that too in people from all age-group categories.^15^ A number of studies have been done in different settings to estimate the prevalence of depression and it has varied from 8.3% in China to 25% in India to 15.4% in Spain.^16^–^18^ All these studies were done in the community settings among the general population using different tools.^16^–^18^ We must acknowledge that different scales or cut-off values have been used to identify the presence of mental health illness among the COVID-19 patients, nevertheless there is a definite association of the infection with being not mentally well.

COVID-19 and Depression among healthcare workers

In a systematic review, the prevalence of depression was estimated to be 24.3% among the healthcare workers who were employed in the care of the COVID-19 patients. Further, it was reported that the prevalence of depression was higher among doctors when compared with other hospital personnel.^19^ The findings of different systematic reviews & meta-analysis revealed that the prevalence of depression and other psychiatric illnesses was very high among the health care professionals.^20^,^21^ These results were primarily due to the stress and anxiety encountered by them in their workplace, including the fear of acquisition of infection or acting as the source of infection to their own family members.

The very fact that patients are losing their lives to the infection by sudden deterioration, and to which no cure is available, accounts for an emotional and mental fatigue.^19^,^20^ The scenario is further compounded by increased workload, physical exhaustion, shortage of personal equipment, the very fact of nosocomial infection & cases being reported among health professionals, makes the overall situation quite challenging.^20^–^22^ It is more of an ethical dilemma, wherein the healthcare workers are expected to discharge their responsibilities, while compromising their own safety. Further, they are subjected to social isolation, lack of social support and prolonged duration of working hours, and thus are vulnerable to multiple mental health problems.^21^–^23^

## Public health interventions

On the community scale, there is an immense need to calm the general population and that can be significantly accomplished by providing the correct information to the masses through authentic sources. The idea is that people should not become victims of myths and understand what needs to be done to overcome the psychological pressures.^24^ There is a definite scope to ensure provision of timely and tailored mental conditioning services by establishing dedicated hotlines and multidisciplinary teams.^25^ These activities will aid in building public security and will ensure psychological benefits. It is important to spread positive vibes among the masses through different channels.^24^,^25^

From the perspective of health care professionals, the need of the hour is to improve their psychological resilience and strengthen the overall functioning of the health system. This can be accomplished by having clear communication with the health staffs, defining the work timings, provision of rest during working shifts, formulation of policies to ensure infection control & promote safe handling of patients, and ensuring the adequate availability of personal protective equipment.^25^,^26^ Further, the use of electronic devices in providing counseling can be also explored to minimize psychological issues.^27^ Moreover, the practice of self-guided interventions and regular physical exercise also plays an important role in minimizing the depression and anxiety attributed to COVID-19 pandemic.^21^,^28^,^29^

## Implications for practice

Considering the ongoing development and the impact of COVID-19 infection on the mental health of people, there is an immense need to implement strategies to improve the lifestyle of the general population and the health care professionals. Steps can be taken to ensure regular monitoring of the mental well-being and the condition can be controlled through prompt interventions. People should be explained about the mind relaxing ways like meditations, listening music, pursuing their hobbies and avoiding consumption of alcohol while being at home. The media has to be more responsible and refrain from spreading rumors about the infection. At the same time, the media should divert their attention towards creating awareness about mental health issues and motivate people to seek medical attention to avoid delay in diagnosis.

## Implication for research

The rise in the incidence of mental health problems, including depression various age-groups and across the globe has become a cause of concern for the public health authorities and the policy makers. The researchers have realized the need to urgently respond to the mental health issues in all the vulnerable population groups. The findings of different systematic review and meta-analysis has revealed that the general population as well as the health care professionals is very much prone for mental trauma amid the development of the disease. On the research front, there is a need to conduct research estimating the prevalence of the mental health issues, including depression among different population sub-groups.

The generated evidence can be used by the policy makers and public health authorities to take evidence-based decisions. The conduction of a cohort study or a randomized control trial will provide better quality evidence and can play an important role in minimizing the magnitude of the illnesses. In addition, we can also conduct studies to identify the impact of specific interventions in reducing the incidence or preventing the progression of mental illnesses.

## Strengths and Limitations

The strength of the review is that COVID-19 has emerged as a global public health emergency for more than a year and continues to account for the sufferings and death of the millions of people across the world. The COVID-19 pandemic has resulted in a sudden rise in the incidence of mental health disorders and in the current review, a large number of studies have been included to understand the impact of the novel viral infection. However, as the literature search was limited to a single search engine, the findings from across the globe have not been compiled.

## Conclusion

To conclude, the ongoing COVID-19 pandemic has created a state of public health emergency on the global scale. The infection has impacted people from all walks of life and is also responsible for precipitating a number of psychological and mental disorders. Thus, it is the need of the hour to identify those individuals who are prone to psychological disorders, and take urgent steps to ensure the preservation and improvement of the mental health of people.
